# The Effect of Feeding Restriction on the Microbiota and Metabolome Response in Late-Phase Laying Hens

**DOI:** 10.3390/ani11113043

**Published:** 2021-10-24

**Authors:** Clara Ajeng Artdita, Yi-Ru Zhuang, Tzu-Yu Liu, Chih-Yuan Cheng, Felix Shih-Hsiang Hsiao, Yuan-Yu Lin

**Affiliations:** 1 Department of Animal Science and Biotechnology, Tunghai University, Taichung City 407, Taiwan; d09610702@thu.edu.tw (C.A.A.); jane890325@gmail.com (T.-Y.L.); jimmy890322@hotmail.com.tw (C.-Y.C.); 2 Department of Bioresources Technology and Veterinary, Vocational College, Universitas Gadjah Mada, Yogyakarta 55281, Indonesia; 3 Department of Animal Science and Technology, National Taiwan University, Taipei City 106, Taiwan; joyzoezu@gmail.com

**Keywords:** dietary restriction, laying hens, serum, cecal bacterial profile, metabolite profile

## Abstract

**Simple Summary:**

Feeding restriction (FR) is essential to reduce excessive fat deposits caused by overfeeding in hens and to ensure their reasonable nutritional requirements for egg production. Effective FR is particularly crucial for raising hens in the late phase of laying; because hens require lower energy at this stage, overfeeding reduces their feed efficiency and increase feed costs. The gut microbiota is involved in various metabolic pathways of laying hens, including in late-phase age. Thus, changes in feeding interventions can alter the presence of gut microorganisms and the structure of the microbial community, resulting in altered metabolic regulation. In this study, we investigate the microbiota and metabolome responses of late-phase laying hens under FR. Our results provide data to access the profile of the cecal bacteria community, their relevance to cecal and serum metabolites, and their FR biosynthetic pathways related to host nutritional requirements and intestinal nutrient availability. Moreover, understanding the principles of host-microbial interaction is essential for developing cost-effective strategies to improve laying hens’ production.

**Abstract:**

This study investigated cecal bacterial community profile, cecal and serum metabolites, and its biosynthesis pathway in late-phase laying hens during 6 weeks feeding restriction (FR), using 16S rDNA as gene sequencing and non-targeted LC-MS/MS as metabolomics approach. We used three groups (ad libitum, FR20, and FR40). FR can reduce excessive fat in late-phase laying hens, while egg production rate is not affected, except for the FR40 group. In phylum level, FR20 had more population of Bacteriodetes and Firmicutes amongst groups. The same result is at genus level, FR20 were higher of the predominant genus (*Bacteroides* and *Rikenellaceae_RC9_gut_group*). Both of FR20 and FR40 reduced Proteobacteria as potential pathogenic bacteria. Non-targeted metabolomic analysis revealed that FR20 modified 20 metabolites in cecal and 10 metabolites in serum of laying hens, whereas 48 cecal metabolites and 31 serum metabolites has revealed in FR40. KEGG assay showed FR20 and FR40 upregulated lipid, carbohydrate, amino acid, nucleic acid pathway, and FR40 modified steroid metabolism in cecal analysis. In serum, only FR40 modified lipid, amino acid pathway, and carbohydrate biosynthesis were shown. This study showed that FR during late-phase laying hens altered the microbiome composition, modified metabolites profile and biosynthesis of the cecal as well as serum.

## 1. Introduction

The intestinal microbiota, the extremely large number of different microorganisms that inhabit the gut, is exclusively responsible for intestinal morphology, nutrient absorption, immunity, and host health, including growth performance [1,2]. This microbiota is also involved in important metabolic functions and numerous host pathways, such as the biosynthesis of lipid and amino acid [1,3]. Therefore, nutritional intervention can induce the presence of intestinal microorganisms [4,5] and the structure of the microbial community [5], which, in turn, affects host immunity and metabolic regulation [6].

The means of nutritional intervention for chicken is called feeding restriction (FR) [7]. In the laying hens industry, FR is an important feeding method to increase egg production of hens [4]; for example, to artificially control the feed intake [7,8], the protein and/or energy levels that are applied to chickens are limited to reduce feed costs in the hen’s diet [9,10]. The purpose is to ensure that the hens will not accumulate too much fat to affect their production performance [11,12,13,14]. Specifically, FR imposed in the rearing stage of the hens can properly control their body weight, avoid premature maturity, and reduce mortality [9,12]; additionally, the number of heavy follicles in the hens will be decreased at onset of the egg production [15]. Before the peak of egg production, FR is usually beneficial to hens for egg production; it has a longer-lasting effect on egg size and egg production capacity and lower mortality in the hens, but this effect may depending on the species of hens and the level of FR used [13]. Although there are few associations between FR and egg production after peak production [16,17], the FR carry out on the hens at this stage has always been the subject of much commercial interest, because hens that reach body maturity at late stage of laying cycle require less dietary energy [18,19]; therefore, applying FR to hens can improve the feed efficiency and save feed costs [16,20].

Gut microbiota has a role in a variety of metabolic processes in laying hens, including late-phase age. As a result, interventions in dietary treatments can modify the presence of gut microbes and the structure of the microbial community, resulting in changes in metabolic control. FR in laying hens can not only alter the population of intestinal microbes [4,21] and its microbial gene expression [5] but also affect host health [4,22] and metabolite regulation. In addition, due to host-related physiological adaptations, chickens with restricted feeding often exhibit different gut characteristics (e.g., lower ileal and cecal short chain fatty acid (SCFA) profiles, bigger duodenum, and enlarged pancreas size), possibly concerning enhancing the utilization of feed to obtain the greater energy and nutrient requirements resulting from FR [4]. This occurs as a form of compensatory dietary restriction, thereby reducing the availability of intestinal nutrients. Moreover, FR is associated with differences in gut bacteria [4,21]. These changes affect the small intestinal tract, possibly giving the microbiota more time to utilize non-digested feed [4]. Accordingly, bacteria that have functional abilities to degrade nondigestible carbohydrates [21] were enriched in the ileum in chicken with FR. Another underlying mechanism might have been a greater mucus secretion to facilitate the ingesta flow in chicken with FR [4].

Recently, metabolomics has become an emerged technique that focuses to identify the functional correlation between the host and the intestinal commensal microbiota. Most studies on the intestinal microbiome aim to understand disease-related metabolites or their dysregulated metabolic pathways [2,23]. This approach is effective for assessing the effect of nutritional interventions, especially when traditional hypothetical methods cannot detect metabolic changes, because they solely focus on nutrient content to maintain a population over improving health and performance [24].

The commensal bacterial community in the intestine is important for chicken metabolism. These bacteria not only interact amongst their community, but also interact with the host tissue [1]. This interaction is fundamental for poultry production and health, because these bacteria can protect the intestine from pathogens [1,25]. Layer production starts to fall after 31 weeks of age [26], and due to aging, the egg productivity and immunity of laying hens decline sharply, and hence can affect the metabolism and hormonal status of these hens [27]. Due to the FR in the late laying period, the changes in hen performance, egg production, and cecal microbial community and its metabolite-microbial community, as well as metabolite in serum, have not been discovered before. Herein, we revealed the cecal bacterial community profiles, cecal and serum metabolites profile, and the correlation of cecal microbiome with metabolites (cecal and serum) and its biosynthesis pathway in late-phase laying hens.

## 2. Materials and Methods

### 2.1. Ethical Statement

All research was approved by the Tunghai University Institutional Animal Care and Use Committee (IACUC Approval No. 106-15) prior to the start of data collection.

### 2.2. Animals

All experiments were performed in accordance with approved guidelines. The animal protocol was approved by the Tunghai University Institutional Animal Care and Use Committee (IACUC Approval No. 106-15) prior to the start of data collection. A total of 30 healthy, 48 week-old Lohmann laying hens from the same hatch of local commercial hatchery were weighed and randomly allotted to one of the three groups at Tunghai University experimental farm located in Taichung, Taiwan. The hens were reared in galvanized wire cages (25 × 40 × 30 cm, one hen per cage) with a nipple drinker and individual trough-feeder. The lighting schedule was 16 h light, 8 h dark throughout the experiment. Mean ambient temperature was 25 ± 5 °C; the relative humidity was maintained within the range of 60–70%. Feed and water were provided ad libitum over the entire experimental period. All hens were provided with the same diet, which was formulated according to the recommendations of National Research Council [28], as shown in Table 1.

### 2.3. Experimental Design

At 48 weeks of age, 30 laying hens were allocated randomly into three experimental groups; each group contained 10 repetitions of 1 hen in one replicate. The three groups were (1) ad libitum (AL) as control group, (2) 20% feed restriction group (FR20), and (3) 40% feed restriction group (FR40). The experiments were carried out over a total of a 6 week period. Before the beginning of data collection, the hens were adapted for two weeks. The AL group consisted of a supply of 100 g of the laying diet per bird a day (Table 1), and the other FR group consisted of a supply of 80 g (FR20) and 60 g (FR40) of laying diet per bird a day, compared to the AL group, respectively. The body weight and egg production in each treatment were recorded daily, and the egg weight was recorded every two days. The dead hens were replaced by spare birds maintained under identical treatment.

### 2.4. Sample Collection

At the end of the experiment, 5 chickens were randomly selected from each group, and cecal contents samples and blood samples were collected for further microbiome and metabolome analysis. Specifically, the hens were injected intravenously with sodium pentobarbital (30 mg/kg body weight), and cervical dislocation was performed. Approximately 2 g of cecal contents were collected, aliquot into two sterilized tubes, and stored at −80 °C. One of these cecal content samples was used for DNA extraction and pyrosequencing, and the other samples was used for metabolomics analysis. Blood was collected from the left brachial vein of the hens; these blood samples were centrifuged at 1500× *g* for 15 min at 4 °C to collect serum. The serum sample was stored at −80 °C for metabolomic analysis. Liver and abdominal fat were collected for weight determination.

### 2.5. DNA Extraction and 16S rDNA Amplicon Pyrosequencing of Feces Sample

The total bacterial genomic DNA in each sample of intestinal contents sample was extracted using QIAamp Fast DNA Stool Mini Kit (QIAGEN, Hilden, Germany). The extracted DNA was then measured using a SimpliNano spectrophotometer (Biochrom, Cambridge, UK) and agarose gel electrophoresis. The paired-end 2 × 300 bp sequencing was performed using the Illlumina MiSeq platform with MiSeq Reagent Kit (Illumina, San Diego, CA, USA).

### 2.6. Sequence Analysis

Quantitative insights into microbial ecology (QIIME, v1.8.0) pipeline was used to process the sequencing data, as described previously [29]. Briefly, we used FLASH v.1.2.11 for assembling the 300 bp paired-end raw reads derived from the 16S ribosomal amplicon sequencing and barcode identification for de-multiplexing. We discarded a Q score of less than the threshold (Q < 20) in the QIIME 1.9.1 pipeline (as a quality control). Before operational taxonomic unit (OTU) clustering at 97% sequence, identified using the UPARSE function in the USEARCH v.7 pipeline, effective tags were filtered and obtained by UCHIME to investigated chimera sequences. Based on the information retrieved from the Silva database v.132, we used an RDP classifier (v.2.2) algorithm to annotate taxonomy classification for each representative sequence. Alpha diversity, based on 6 criteria from QIIME pipeline (i.e., observed-OTU, Chao1, Shannon, Simpson, abundance-based coverage estimators (ACE), and good-coverage), indicate the complexity of each species within individual samples. Meanwhile, the number of different species represented in the microbial community is referred to as observed-OTU. The Chao1 and ACE indices was used for investigating community richness, and the relative abundance and evenness accounting for diversity were evaluated by the Shannon indices. For representing the number of the observed species, a random selection of a certain amount of sequencing data of each sample was used to construct a rarefaction curve. We then analyzed the differences among samples in terms of species complexity using beta diversity. A principal component analysis (PCA) preluded a cluster (genus) analysis whose function was to reduce the dimensions of the multiple variables using the FactoMineR package and ggplot2 package in R software (v.2.15.3). For enhancing the community distinction, the partial-least-squares discriminant analysis (PLS-DA) was used to analyze and visualize variance based on OTU level of gut microbiota composition among the communities. This PLS-DA can be evaluated using the R package mixOmics. For statistical analysis, a zero-inflated Gussian (ZIG) log-normal model, as implemented in the “fitFeatureModel” function of the Bioconductor metagenomeSeq package, was used to determine the significance of all species among groups at various taxonomic level, as previously described [29]. Furthermore, to determine whether the community structures significantly differ among and within groups, we then used analysis of similarities (ANOSIM) analysis. This analysis also provided microbial phenotype, and this database source could be revealed in the integrated microbial genomes (IMG) database, the Kyoto Encyclopedia of Genes and Genomes (KEGG) and the Pathosystems Resource Integration Center (PATRIC) [30,31]. BugBase was used to predict organism-level microbiome phenotypes; specifically, BugBase uses input from various databases, including IMG, KEGG, and PATRIC, to categorize six main phenotype categories: Gram staining, oxygen tolerance, ability to form biofilms, mobile element content, pathogenicity, and oxidative stress tolerance [32].

### 2.7. Metabolomic Extraction of Feces and Serum Sample

We used 100% methanol extraction method (methanol, with 1 μg/mL internal standard) to obtain metabolites from the cecal contents and blood serum. Briefly, a hundred microliter of serum or sample of cecal contents was mixed with 400 μL of extraction solution in an eppendorf tube. The samples were sonicated in an ice-water bath for 10 min and then incubated at −20 °C for 1 h to precipitate protein. These extracted samples were then centrifuged at 15,000× *g* for 25 min at 4 °C. Afterwards, each supernatant was transferred to a fresh glass vial for LC–MS/MS analyses. A quality control (QC) sample was then prepared by mixing aliquots of supernatants from all samples.

### 2.8. Metabolomic Analysis

The cecal contents of LC–MS/MS analyses were determined using an UHPLC system (1290, Agilent Technologies) with a UPLC HSS T3 column (2.1 mm × 100 mm, 1.8 μm) coupled with a Q Exactive mass spectrometer (Orbitrap MS, Thermo). The mobile phase A contained 0.1% formic acid in water (as positive mode) and 5 mmol/L ammonium acetate in water (as negative mode), and the mobile phase B contained acetonitrile. Next, the elution gradient was set: 1% B for 0–1.0 min, 1–99% B for 1.0–8.0 min, 99% B for 8.0–10.0 min, 99–1% B for 10.0–10.1 min, and 1% B 10 for 1–12 min with 0.5 mL/min of flow rate. The QE mass spectrometer was employed due to its ability to acquire MS/MS spectra on the information-dependent acquisition (IDA) mode in the control of the acquisition software (Xcalibur 4.0.27, Thermo). Furthermore, the ESI conditions were set as follows: capillary temperature was at 400 °C, full MS resolution was 70,000, sheath gas flow rate was 45 Arb, Aux gas flow rate was 15 Arb, MS/MS resolution was 17,500, collision energy was 20/40/60 in NCE mode, and voltage spray was 4.0 kV (positive) or −3.6 kV (negative), respectively.

### 2.9. Metabolomic Data Processing

After LC–MS/MS analysis, ProteoWizard was used to convert the acquired raw data into mzXML format and to process with an in-house program, which was developed using R and based on XCMS for peak detection, extraction, alignment, and integration. The normalized total peak intensity was then analyzed by multivariate data, including PCA and orthogonal partial least squares discriminant analysis (OPLS-DA). The importance of each variable in the OPLS-DA model in the projection (VIP) value was further calculated to show its contribution to the classification. Metabolites with a VIP score > 1 and a *p* value less than 0.05 are considered statistically significant. The functional interpretation of the relevant KEGG pathways in these metabolites was revealed by the MetaboAnalyst, based on hypergeometric testing [33].

### 2.10. Statistical Analysis

Statistical analysis was performed using GraphPad software (version 5 for Windows). Significant differences between each treatment were measured using *t*-test, and *p* < 0.05 was considered significant. The asterisks (*) represent the statistically significant difference in the production performance of hens with different FR programs (FR20 or FR40) compared to the AL group (*p* < 0.05). Differences were considered significant for *p* values of 0.05 and were considered to represent trends for *p* values greater than 0.05 and less than or equal to 0.10.

## 3. Results

### 3.1. Effect of FR on Production Performance of Laying Hens

**Figure 1:**
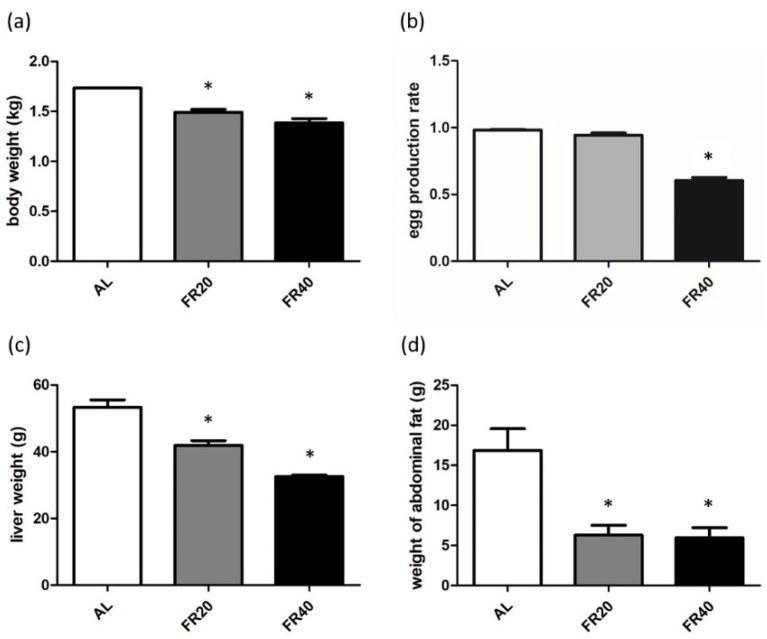
Effect of FR on production performance of laying hens: (**a**) body weight; (**b**) egg production; (**c**) liver weight; (**d**) abdominal fat between AL, FR20, and FR40 group. AL: ad libitum with basal diet; FR20: basal diet with 20% feed restriction; FR40: basal diet with 40% feed restriction. * *p* < 0.05 comparing data of FR20 or FR40 versus AL group.

Figure 1a showed that FR significantly reduced the body weight of FR20 and FR40 treated hens compared to the AL group (*p* < 0.05). Similar with the body weight, the weight of liver, and abdominal fat in laying hens was also affected by the increase in FR. Specifically, restrictive feeding significantly reduced the weight of liver and abdominal fat in FR20 and FR40 treatment compared to ad libitum feeding (*p* < 0.05, Figure 1c,d), while FR40 had a significant reduction in liver weight compared to FR20 (*p* < 0.05, Figure 1c). Although there was no significant difference in the egg production rate between FR20 and AL group (Figure 1b), the egg production rate was significantly reduced in FR40 treatment (*p* < 0.05, Figure 1b). However, there is no significant difference between the FR20 egg production level and AL group (*p* < 0.05, Figure 1b), which indicated that the egg production efficiency of hens can be improved under the condition of moderate feed restriction [13,22].

### 3.2. Microbiota Sequencing

**Figure 2:**
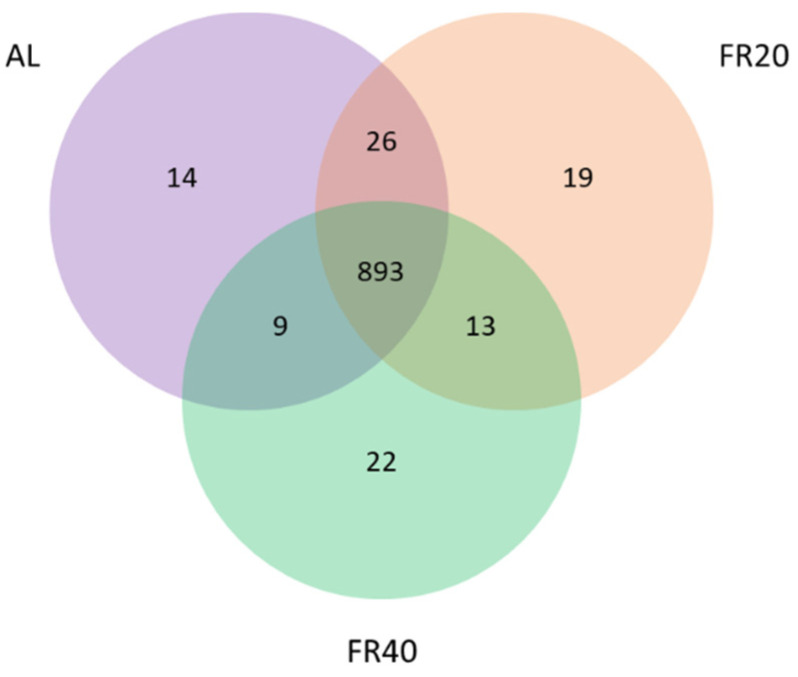
Venn diagram analysis of 996 OTU in the cecum of hens. Venn diagram showed 893 OTU among these three groups. A total of 14, 19, and 22 OTU were found in AL, FR20, and FR40 groups, respectively. Furthermore, 26 OTU were found in the AL and FR20, 13 OTU were found among the FR20 and FR40, and 9 OTU were found in the AL and FR40 groups. AL: ad libitum with basal diet; FR20: basal diet with 20% feed restriction; FR40: basal diet with 40% feed restriction.

After quality trimming and chimera checking through the QIIME pipeline, effective tags were revealed in each sample, and operational taxonomic units (OTU) were picked by clustering at the 97% identity level using UPARSE. Moreover, Venn diagram analysis revealed a total of 996 OTU in the cecum of hens. Among them, 14, 19, and 22 OTU were found in AL, FR20, and FR40 groups, respectively. In addition, in the AL and FR20, FR20 and FR40, and AL and FR40 groups, 26, 13, and 9 OTU were found as common OTU, respectively. Further, Venn diagram showed 893 OTU among these three groups (Figure 2). Species-accumulation curve analysis [34] was then used to estimate the number of species in these samples. It was revealed that a large proportion of species existed in the cecal community (Figure S1). In particular, we showed a sharp increase in the measurement curve, which means there was a substantial increase in species abundance. In addition, the flat curve showed that the number of species did not increase, even though the number of samples increases. The results of this increase in the number of samples indicated that the samples number provided in this study is sufficient.

### 3.3. Effect of FR on Microbial Diversity and Relative Abundance

**Figure 3:**
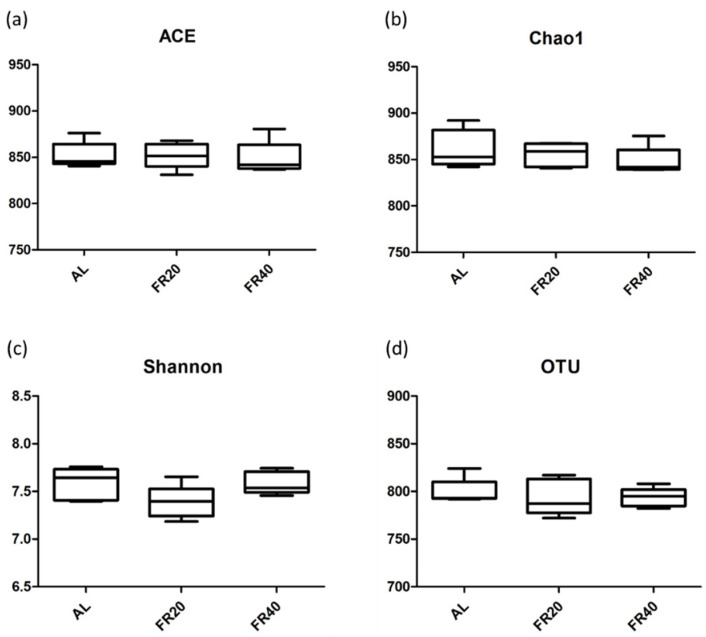
Differences in bacterial community diversity and richness in the cecum of laying hens among AL, FR20, and FR40 group. The community richness estimates of (**a**) ACE and (**b**) Chao1, the diversity indices of (**c**) Shannon, and (**d**) the OTU numbers are shown. AL: ad libitum with basal diet; FR20: basal diet with 20% feed restriction; FR40: basal diet with 40% feed restriction.

**Figure 4:**
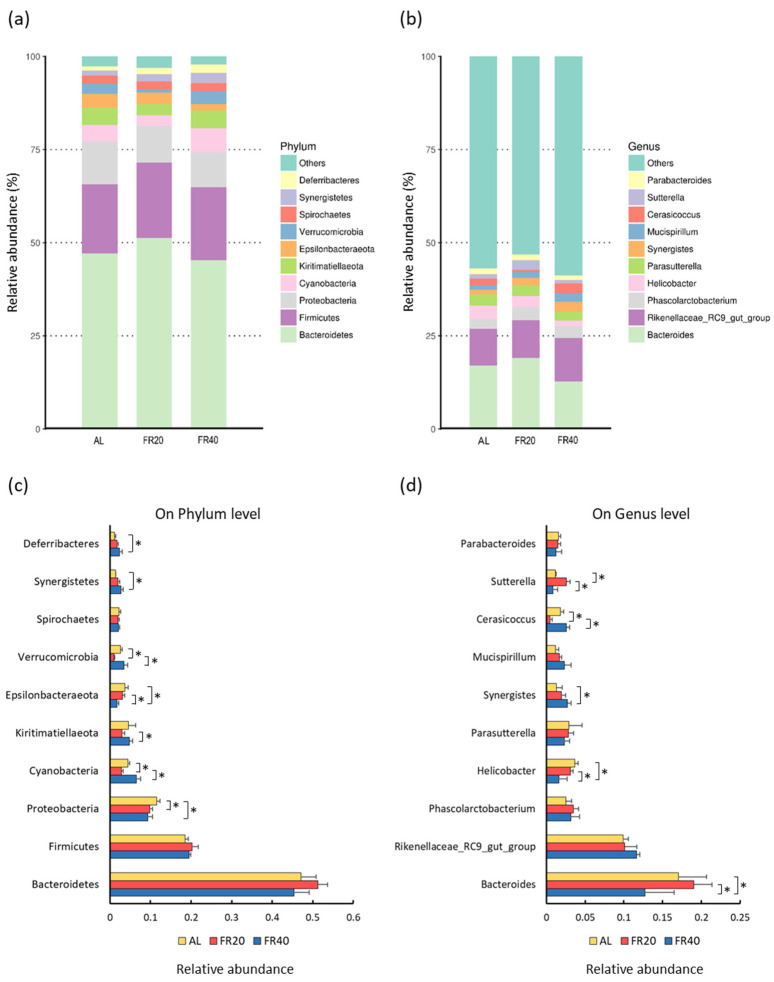
Composition of the top 10 microorganisms in the cecum of the hens among normal feeding and restriction feeding group at (**a**) phylum level and (**b**) genus level. A comparison of the relative abundance (percentage of sequence) of (**c**) the main bacterial phyla and (**d**) the main bacterial genera is shown. The calculation is based on the average relative abundance of the main bacterial communities. * *p* < 0.05 compare the data between FR20 and 40 and FR20 or FR40 and AL group. AL: ad libitum with basal diet; FR20: basal diet with 20% feed restriction; FR40: basal diet with 40% feed restriction.

Chao1, ACE, and Shannon are several indices that calculate the abundance or distribution of OTU within a particular population. For example, low richness suggests a low number of species in the community, and low evenness indicates that the sample consists of a few dominating taxa. Chao1, ACE, and OTU-observed species indices are indicators for species richness analysis of gut microbiome; OTU also can classify a group of closely related species [35], while Shannon is used to predict species diversity of a community [35,36]. The diversity of cecum microbiota in three groups, AL, FR20, and FR40, is shown in Figure 3. Compared to the AL group, the restrictive feeding treatments (FR20 and FR40) of the hens had no significant difference in Chao1, ACE, and OTU-observed species indices (Figure 3a,b,d, respectively). A similar result appeared in Shannon, where restrictive feeding did not affect the diversity of species in FR20 and FR40, as compared to AL group (*p* < 0.05, Figure 3c). Nevertheless, FR resulted in a change in the cecum microbial composition. However, restrictive feeding resulted in changes in the microbial composition of the cecum. Phylogenetic classification of OTUs (Figure 4a) revealed that Bacteroidetes and Firmicutes were the predominant phyla. The relative abundances of Bacteroidetes in FR20 and FR40 were 51.29% and 45.32%, respectively, compared to the AL group (47.08%). Similar to Bacteriodetes, the relative abundances of Firmicutes were higher in the FR group (FR20 was 20.21% and FR40 was 19.52%) than in the AL group (18.57%). On the other hand, the relative abundances of Proteobacteria were higher in the AL group (11.57%) than in both the FR20 group (9.83%) and FR40 group (9.34%); these findings are similar to the abundance of two phyla, Epsilonbacteraeota (AL, FR20, and FR40 were 3.73%, 3.13%, and 1.70%, respectively) and Spirochaetes (AL, FR20, and FR40 were 2.24%, 2.00%, and 2.1%, respectively). Furthermore, the relative abundance of Cyanobacteria (6.46%), Kiritimatiellaeota (4.85%), Verrucomicrobia (3.5%), Synergistetes (2.72%), and Deferribacteres (2.34%) were higher in FR40 than in FR20 and AL. We also predominantly compared the relative abundance of genera (Figure 4b) with the level reached by *Bacteroides* and *Rikenellaceae_RC9_gut_group*. FR20 (19.06%) showed the higher relative abundance of genus *Bacteroides* followed by AL (17.01%) and FR40 (12.73%). In *Rikenellaceae_RC9_gut_group* relative abundance, the FR40 group (11.67%) was higher than in the FR20 group (10.11%) and AL group (9.9%). The three genera, *Helicobacter* (3.69%), *Parasutterella* (2.93%), and *Parabacteroides*, (1.54%)) of the AL group were higher than in both FR treatments, FR20 (*Helicobacter* (3.1%), *Parasutterella* (2.86%), and *Parabacteroides* (1.5%)) and FR40 (*Helicobacter* (1.6%), *Parasutterella* (2.29%), and *Parabacteroides* (1.27%)). Beyond the genus *Bacteroides*, FR 20 was also higher in *Phascolarctobacterium* (3.47%) and *Sutterella* (2.59%) compared to FR40 (*Phascolarctobacterium* and *Sutterella* was 3.14% and 0,84%, respectively). In particular, FR40 showed a higher result in three genera (*Synergistes* (2.73%), *Mucispirillum* (2.34%), and *Cerasicoccus* (2.61%)) compared to FR20 (*Synergistes* (1.94%), *Mucispirillum* (1.7%), and *Cerasicoccus* (0.44%)) and AL (*Synergistes* (1.33%), *Mucispirillum* (1.16%), and *Cerasicoccus* (1.81%)). *Parabacteroides* slightly decreased in FR40 (12.68%) compared to FR20 (15.13%) and AL (15.39%).

Furthermore, we also investigated the details of the main phylum (Figure 4c) and main genus (Figure 4d). In the main phylum (Figure 4c), although Bacteroidetes and Firmicutes were the predominant phyla, there was no significant difference amongst the group. The AL group has a higher relative population of Proteobacteria with significant differences compared to FR20 and FR40 (*p* < 0.05). The relative abundance of Cyanobacteria, Kiritimatiellaeota, and Verrucomicrobia of FR40 were significantly higher than FR20 (*p* < 0.05). However, the AL group was significantly increased in Cyanobacteria and Verrucomicrobia compared to FR20 (*p* < 0.05). FR40 significantly reduced the relative abundance of Epsilonbacteraeota compared to FR20 and AL (*p* < 0.05). There was no significant difference in phylum Spirochaetes amongst the group. In addition, Synergistetes and Deferribacteres significantly increased in FR40 compared to in the AL group (*p* < 0.05). Moreover, at the genera level (Figure 4d), the most predominant genus was *Bacteroides*, and the FR40 group decreased its relative abundance compared to the FR and AL groups (*p* < 0.05). Four genera (*Rikenellaceae_RC9_gut_group*, *Phascolarctobacterium*, *Mucispirillum*, and *Parabacteroides*) had no significant difference amongst the group. FR20 significantly increased in *Helicobacter* and *Sutterella* compared to FR40 (*p* < 0.05). In *Helicobacter*, FR40 also decreased compared to the AL group (*p* < 0.05), and in *Sutterella*, AL was lower than FR20 (*p* < 0.05). Nevertheless, AL and FR40 were sharply increased in *Cerasicoccus* compared to FR20 (*p* < 0.05).

**Figure 5:**
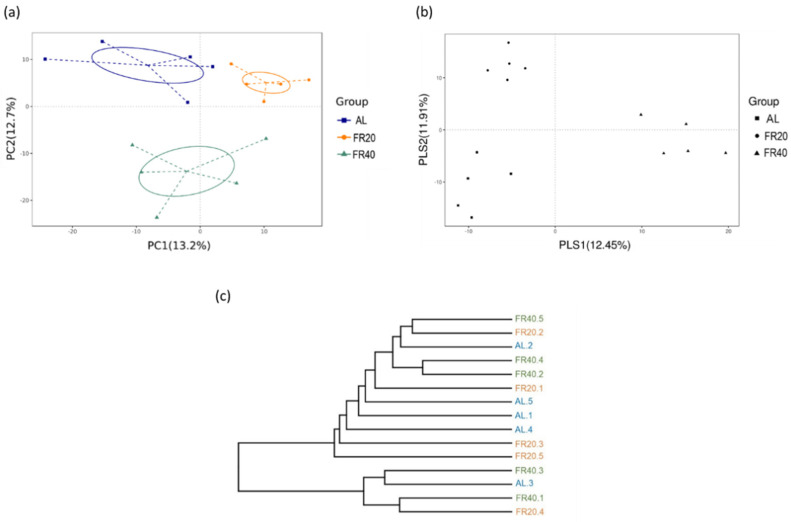
The similarity of the identified OTU among each group: (**a**) Principal components analysis (PCA) of the bacterial community structure among AL (square—blue color), FR20 (circle—orange color), and FR40 (triangle—green color). Each symbol represents each gut microbiota; (**b**) partial least squares discriminant analysis (PLS-DA). Each symbol represents each gut microbiota. Square symbol represents AL group, circle symbol represents FR20, and triangle symbol represents FR40; (**c**) Simple agglomerative hierarchical clustering method based on WPGMA. AL: ad libitum with basal diet; FR20: basal diet with 20% feed restriction; FR40: basal diet with 40% feed restriction.

Principal component analysis (PCA) was used to investigate the distribution of identified OTU (microbiota) in each current taxon. Our result showed that the clusters between the treatments (AL, FR20, and FR40 groups) were significantly separated (*p* < 0.05, Figure 5a); the principal components PC1 and PC2 had 13.2% and 12.7% variation, respectively. Further, partial least squares discriminant analysis (PLS-DA) was used to reveal the group distinction, indicating that the identified OTU had a good discrimination between groups (*p* < 0.05, Figure 5b); the variation of PLS1 was 12.45%, and that of PLS2 was 11.91%. These results indicated that PCA and PLS-DA could clearly distinguish the OTU revealed in this study; the OTU in the microbial population were different between the AL, CR20 and CR40 groups.

We also explored the similarity of the identified OTU among each group. To this end, a simple agglomerative hierarchical clustering method based on a weighted pair group method with arithmetic mean (WPGMA) was first used to create clusters of similar origin in the treatment (AL, FR20 and FR40 groups; Figure 5c). Then, a statistical test with ANOSIM was performed to examine whether there was a significant difference between the groups. Of these, the *R* value of the FR20 and FR40 groups was 0.456, while the Bonferroni corrected *p* value was 0.008. The *R* value of the AL and FR40 group was 0.536, and the Bonferroni corrected *p* value was 0.01. The AL and FR20 groups resulted in an *R* value of 0.208 with a Bonferroni corrected *p* value of 0.017 (Table S1), suggesting that there was significant dissimilarity among the different groups.

**Figure 6:**
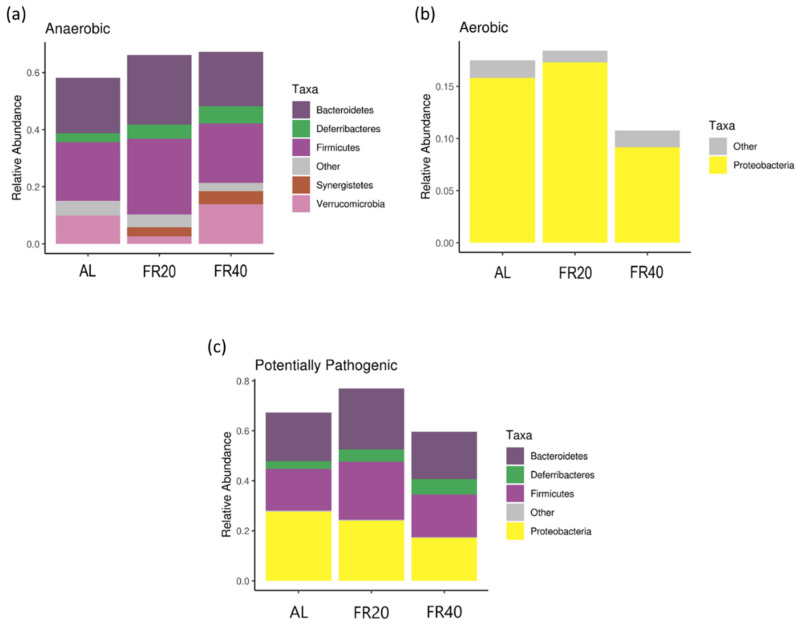
Microbial phenotype in cecum: (**a**) anaerobic microbiota; (**b**) aerobic microbiota; and (**c**) pathogenic microbiota. AL: ad libitum with basal diet; FR20: basal diet with 20% feed restriction; FR40: basal diet with 40% feed restriction.

The ontology of the microbial phenotype was then revealed. Importantly, as compared to AL group, the restricted feeding group represented higher anaerobic bacteria and less aerobic flora (*p* < 0.05, Figure 6a,b). The three groups have the same number of anaerobic bacteria that dominate the cecum (Firmicutes and Bacterioidetes), with FR20 having the higher number (*p* < 0.05, Figure 6a). In addition, Proteobacteria were the only aerobic bacteria that dominate the cecum among the three groups (*p* < 0.05, Figure 6a). In this analysis, FR20 has highest number amongst all other groups (*p* < 0.05, Figure 6b). Moreover, the FR40 group observed a decrease in the total population of potential pathogenic bacteria (*p* < 0.05, Figure 6c). Both FR20 and FR40 reduced Proteobacteria as potential pathogenic bacteria (*p* < 0.05, Figure 6c), indicating that the phenotype of the intestinal microbiota may be affected by FR in laying hens.

### 3.4. Effect of FR on KEGG Pathway in Cecum and Serum

An untargeted LC–MS-based metabolomics platform was used to analyze the cecum contents and the serum metabolite profiles of chicken fed ad libitum and restricted. According to the variable importance in the projection (VIP) value > 1, in 95% jack-knifed confidence intervals and *p* < 0.05, detailed information about the different biomarker metabolites has been shown in Table S1. Compared to the AL group, the FR20 group had 20 different metabolites, with 7 LC–MS/MS (ESI+) and 13 LC–MS/MS (ESI−), which were detected in the cecal contents, and 10 different metabolites (8 ESI+ and 2 ESI−), which were detected in serum contents. The FR40 group has 48 different metabolites, with 18 ESI+ and 30 ESI−, which were detected in cecal contents, and 31 different metabolites (16 ESI+ and 15 ESI−), which were detected in serum, as compared to the AL group.

**Figure 7:**
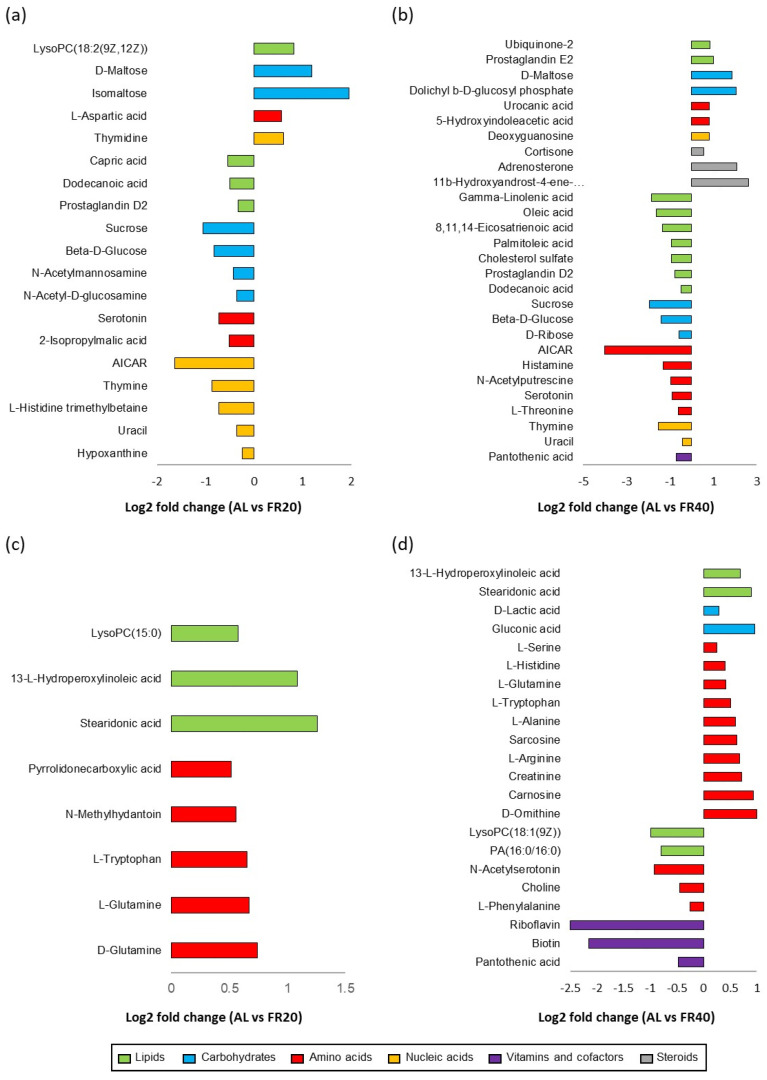
Significantly different metabolites in the cecum and serum of laying hens among normal feeding and restricted feeding group. Different metabolites of AL vs. FR20 in the cecum (**a**), AL vs. FR40 in the cecum (**b**); different metabolites of AL vs. FR20 in the serum (**c**), AL vs. FR40 in the serum (**d**). The accountable metabolites for class discrimination with VIP > 1 and *p* < 0.05 were listed; the web-based pipeline MetaboAnalyst [33] was used to enrich the relevant KEGG pathways. AL: ad libitum with basal diet; FR20: basal diet with 20% feed restriction; FR40: basal diet with 40% feed restriction.

The enrichment of relevant KEGG pathways in these metabolites was further revealed by the web-based pipeline MetaboAnalyst [33]. Figure 7 showed the different metabolites in the cecal and serum contents. As shown in Figure 7a, compared to the AL, the FR20 group modified relevant pathways in one more lipid, two carbohydrates, one amino acid, and one nucleic acid. These metabolites upregulated in cecal contents included LysoPC (18:2(9Z,12Z)), carbohydrate-related metabolites isomaltose and D-maltose, amino acid metabolite L-aspartic acid, and thymidine as the nucleic acid metabolite. Three lipid-, four carbohydrate-, two amino acid-, and five nucleotide-related metabolites were downregulated in cecal contents of chicken with FR20 compared to the AL group; further decreased metabolites were AICAR and thymine (nucleotide-related metabolites), sucrose and beta-D-glucose (carbohydrate-related metabolites), and serotonin (amino acid-related metabolites). Additionally, in FR20, three more lipid-related metabolites (stearidonic acid, 13-L-Hydroperoxylinoleic acid, and LysoPC (15:0)) and five amino acid-related metabolites (D-Glutamine, L-Glutamine, L-Tryptophan, N-Methylhydantoin, and Pyrrolidonecarboxylic acid) were increased in the serum of chicken. There were no downregulated metabolites in this group compared to in the AL group (Figure 7c).

Furthermore, as compared to the AL group, FR40 modified the relevant pathways of two more lipids, two more carbohydrates, two more amino acids, one more nucleotide, and three more steroids, which were increased in cecal contents. These higher metabolites included carbohydrate-related metabolites such as Dolichyl b-D-glucosyl phosphate and D-maltose, steroid-related metabolites including 11b-Hydroxyandrost-4-ene-3,17-dione and adrenosterone, and prostaglandin (E2) as a lipid-related metabolite. Seven lipids, three carbohydrates, five amino acids, two nucleic acids, and one vitamin and cofactor (pantothenic acid)-related metabolites were decreased in cecal contents. These metabolites included AICAR, histamine, sucrose, beta-D-glucose, gamma-linolenic acid, oleic acid, thymine, and pantothenic acid (Figure 7b). In serum contents, as shown in Figure 7d, the most upregulated metabolites in FR40 were amino acids (ten more amino acid), including D-Ornithine, carnosine, creatinine, and L-Arginin. The other metabolites were two lipids (Stearidonic acid and 13-L-Hydroperoxylinoleic acid) and two carbohydrates (gluconic acid and D-lactic acid) as compared to the AL group. Additionally, three more vitamins and cofactors (riboflavin, biotin, and pantothenic acid), two more lipid-related metabolites (LysoPC(18:1(9Z)) and PA (16:0/16:0)), and three more amino acid related metabolites (N-Acetylserotonin, choline, and L-Phenylalanine) were downregulated in serum contents of chicken with FR40 compared to the AL group chicken.

### 3.5. The Relationship of Different Relative Abundance of Bacteria in the Cecal Microbiota with Cecal and Serum Metabolites

Pearson’s correlation analyses showed that the relative abundance of different bacteria (LEfSE) at the phylum level in the cecal microbiota were found to be closely associated with the concentration of specific metabolites in the cecum and serum of chickens (Figure S3). Cecal microbiota of FR20 group showed that Firmicutes were the high proportion of bacteria-correlated metabolites in the phylum level, followed by Bacteriodetes, Proteobacteria, Elusimicrobia, Euryarchaeota, and Verrucomicrobia. Firmicutes were positively correlated with LysoPC(18:2(9Z,12Z)) especially for genus *Oribacterium*; L-Aspartic acid for genus *Ruminiclostridium_9*, *Oribacterium*, and *Butyricicoccus*; beta-D-Glucose metabolite for genus *Ruminococcaceae_UCG_004* and *Ruminococcaceae_UCG_014*; and AICAR metabolite for genus *Ruminococcaceae_UCG_014*, *Ruminococcaceae_UCG_005*, and *Lachnospiraceae_NK4A136_group*. In addition, this phylum was negatively correlated with LysoPC(18:2(9Z,12Z)) for genus *Ruminococcaceae_UCG_014*, *Ruminococcaceae_UCG_004*, *Ruminococcaceae_UCG_005*, *Christensenellaceae_R_7_group*, and *Ruminococcaceae_UCG_010*; Thymidine metabolite for genus *Ruminococcaceae_UCG_004* and *Christensenellaceae_R_7_group*; D-Maltose for genus *Ruminococcaceae_UCG_010*, *Christensenellaceae_R_7_group*, *Ruminococcaceae_UCG_005*, and *Ruminococcaceae_UCG_004*; L-Aspartic acid metabolite for *Ruminococcaceae_UCG_014*, *Ruminococcaceae_UCG_004*, and *Ruminococcaceae_UCG_010*; beta-D-Glucose metabolite only for genus *Ruminiclostridium_9*; and AICAR metabolite only for genus *Oribacterium*. Furthermore, other phylum, Bacteroidetes, had two genus that correlated with cecal metabolites. Genus *Prevotellaceae_Ga6A1_group* was positively correlated with Thymidine and D-Maltose. On the other hand, genus *Rikenella* was negatively correlated with Thymidine and D-Maltose. Furthermore, genus *Azospirillum_sp_47_25* from phylum Proteobacteria had positively correlated with beta-D-Glucose and negatively correlated with LysoPC(18:2(9Z,12Z)) and L-Aspartic acid. Moreover, genus *Elusimicrobium* (phylum Elusimicrobia), genus *Methanocorpusculum* (phylum Euryarchaeota), and genus *Cerasicoccus* (phylum Verrucomicrobia) were negatively correlated with D-Maltose, LysoPC(18:2(9Z,12Z)), and D-Maltose, respectively (Figure S3).

As shown in Figure S4, there was no positively correlation between cecal microbiota of FR20 group and serum metabolites. On other hand, phylum Cyanobacteria, Euryarchaeota, Verrucomicrobia, and Elusimicrobia had negatively correlated with D-Glutamine metabolites but had no significant differences.

The relative abundance of phylum microbiota in the cecal of FR40 group was Fusobacterium (*p* < 0.05) that had positively correlated with oleic acid and negatively correlated with dodecanoic acid (Figure S5). Additionally, microbiota in this group’s cecal, Fusobacterium had positively correlated with choline and negatively correlated with L-Glutamine, L-Arginine_1, L-Histidine, and L-Alanine as metabolites in serum chicken of FR40 group (Figure S6).

## 4. Discussion

### 4.1. Performance Parameters

FR is one of the important methods of feeding and management of commercial laying hens. It is an effective way to control the feed intake to limit the energy and crude protein levels of the hens in the diet so as to improve the performance of layer flock. FR for hens during the rearing period was beneficial in maintaining their proper weight, allowing the bones and internal organs to be fully developed, and avoiding premature maturity [9,12], because these conditions have a negative impact on their egg production performance. In the late phase of the laying hens, due to aging [37], the laying performance (egg production and egg quality) of laying hens decrease quickly [38]; also, the mature body weight causes them to require lesser dietary energy [18,19]. Hence, the main purpose of FR for laying hens during this period is to prevent them from overfeeding and reducing the amount of abdominal fat in the laying hens, so that the laying hens can maintain a proper weight.

It has been indicated that, when the FR reaches 10%, for 54 weeks, laying hens’ egg production rate is not affected [16]. However, for FR20, the egg production rate of hens was significantly reduced. In our study, FR20 was not observed to have a significant effect on the egg production rate of 48 weeks laying hens compared to AL group. Moreover, in the FR40 group, the egg production rate of hens is significantly decreased compared to that of AL control (*p* < 0.05). This result is similar to those of other studies [11,39,40]. It indicated that the egg production efficiency of hens can be improved under the condition of moderate feed restriction [13,22]. According to [39,41], the egg production level of laying hens depends on the energy intake. In this way, the more intense the restriction of energy intake, the greater the negative effect on egg production. Although Snetsinger et al. [42] stated that only 6–7% FR can be used in poultry after 40 weeks to significantly reduce the egg production rate, recent evidence has revealed that the effect of FR on hens’ laying performance may relate to the type of layer used and the implementation of FR [13].

FR program plays an important role in the growth performance and nutrient utilization of laying hens [11]. Consistent with previous reports [16,18,20], we demonstrated that FR could reduce the body weight of hens in the late-phase of laying; additionally, we recorded a decrease in the abdominal fat weight of these hens. This may indicate that the FR levels used in this study are not sufficient to support unwanted fat deposits, which tends to support the suggestion of Kingsley et al. [17], that FR chickens make more efficient use of ingested feed through higher metabolic efficiency and fewer excess fat deposits.

Since FR can reduce the efficiency of liver metabolism, the effect of the intensity and duration of the FR can lead to a reduction in liver weight [43]. Our results showed that, compared with the AL control, FR reduces the liver weight of laying hens, which may further lead to the reduction in abdominal fat in FR20 and FR40; it is correlated to the activity of lipogenic enzyme, which was decreased during the period of FR and gradually declined in the subsequent weeks [44]. In general, healthy laying hens have abdominal fat, as abdominal fat can be an important body resource to maintain the laying when the dietary nutrient supply is insufficient for the egg formation [45]. High levels of abdominal fat may trigger fatty liver degeneration, which leads to negative impacts on body metabolism [46]. Consider the purpose of applying FR to laying hens is to prevent them from overfeeding and reduce the amount of abdominal fat in later laying hens. Therefore, FR measures are beneficial for maintaining the proper weight of laying hens.

### 4.2. Effect of FR on Microbial Diversity and Relative Abundance

In order to design nutritional strategies to effectively improve chickens’ feed efficiency, the relationship between a chicken’s feed intake, intestinal microbiota, and host nutritional metabolism needs to be clarified to better understand the underlying modes of action for the difference in feed efficiency [1,2,47]. Both chicken feed intake and intestinal bacterial microbiota differ between chickens with high and low feed efficiency [4,21]. Additionally, FR is also related to intestine microbiota [4]. Sergeant et al. [48] explained that intestinal microbiota plays a vital role in the health, production performance, and welfare of chickens, including laying hens. The most dense microbiota population in chicken intestine is in the ceca, a pair of blind-ended sacs between the small and large intestines [49]. In addition to the microbiota, the ecological environment in the cecum of laying hens is also composed of metabolites [50]. Thus, there is a correlation between the gut microbiota and feed efficiency in chicken production. We hypothesized that this approach would be helpful in clarifying whether differences in feed intake between hens with high and low feed efficiency (feed restriction) would have an impact on shifts in taxa abundance in the composition of the bacterial community. We observed a strong impact of restrictive feeding on the intestinal physiology and bacterial communities (microbiota), mainly influencing the predominant bacteria (phyla and genera) in cecal and serum.

This study showed that FR increased the relative abundance of phyla Bacteriodetes and Firmicutes, especially for FR20 treatment. This result was consistent with a previous study on FR-enriched phylum Bacteriodetes [50,51] and Firmicutes [4,50,51]. Meanwhile, *Bacteriodes* and *Rikenellaceae_RC9_gut_group* were the major abundant genera in laying hens cecal. A previous study [51] showed that genera *Bacteriodes* and *Rikenellaceae_RC9_gut_group* were dominant in the cecal of laying hens, even though it had heat stress; furthermore, this mainly microbial composition was related to feed intake.

All group experiments investigated the relative abundancy and dominance of anaerobic bacteria. Firmicutes (Gram-positive) and Bacteriodetes (Gram-negative) were relatively abundant in FR20 and FR40 groups; furthermore, Verrucomicrobia and Synergistetes (both Gram-negative bacteria) were less abundant in FR20 than in FR40. The predominant culturable bacteria in the poultry cecal are obligate anaerobes at the level of 10^11^/g of content [52]. Similar to another study [53], Firmicutes, a Gram-positive and obligat anaerob bacteria, is the predominant phylum found in poultry cecal. Meanwhile, Gram-negative non-sporing anaerobic rod bacteria, also found in poultry, duck, and turkey cecal, is of the family Bacteroidaceae [52]. A small proportion of phyla found in chicken intestines included Cyanobacteria, Spirochaetes, Synergistetes, Fusobacteria, Tenericutes, and Verrucomicrobia [54]. In the normal cecal adult chicken, over 40 different types of anaerobic Gram-negative and Gram-positive non-sporing rods and cocci have been found, as have at least 17 different species of Clostridia [55]. There are many other organisms that are still to be isolated and characterized, including Proteobacteria, an aerobic bacteria, found predominantly in chicken cecal after Firmicutes and Bacteriodetes [52,53,54].

The presence of normal flora in the intestine mainly functions to control or eliminate an invading pathogen; it might be considered competition for limiting carbon sources [55]. The pathogen result of these experiment was Bacteriodetes, Firmicutes, and Proteobacteria. Both FR20 and FR40, especially FR40, could decrease Proteobacteria as potentially pathogenic, compared to the AL control. Among the Proteobacteria, the predominant genera were *Desulfohalobium*, *Escherichia*, *Shigella*, and *Neisseria* [56]. One of them, *Escherichia coli*, is an intestine gamma proteobacterium. During the whole life cycle of healthy chickens, this bacterium is found in low abundance. However, several strains of *Escherichia coli* have specific virulence factors may have infected in chickens and cause disease; these strains are known as avian pathogenic *Escherichia coli* (APEC). APEC is principally associated with extra intestinal infections that affect the respiratory tract [54]. This result indicates that FR could decrease the population of potential pathogenic bacteria. Videnska et al. [57] showed that, in the ceca of mature laying chickens (up to 60 weeks old), the representative microbial communities at the phylum level, in order of their typical abundance, are *Proteobacteria*, *Firmicutes*, and *Bacteroidetes*, which formed the vast majority of microbiota across all age categories. This indicated that Gram-negative bacteria were the abundant phyla that dominate the gut, and *Firmicutes* become more dominant in the later age of the laying hen [58].

### 4.3. Effect of FR on KEGG Pathway in Cecum and Serum

Gut microbiota performs a large number of roles of the host through functional microbial pathways. Metabolomic analysis revealed that LysoPC(18:2(9Z,12Z)) upregulated the KEGG pathway of lipid synthesis in the cecal content of the FR20 group. LysoPC(18:2(9Z,12Z)) is Lysophosphatidylcholines, one of the major structural lipids in a eukaryotic membrane cell. Higher phosphatidylcholine demonstrated that the lipid membrane cell structure is under elastic stress. This stress changes a membrane’s physical properties and results in its biological function by modifying the membrane and influencing lipid–protein interaction [59]. This is a normal condition for the membrane to deal with stress, so that it can still control the lipid function of the membrane. Furthermore, at the same location of the FR20 group, isomaltose and D-maltose also upregulated the KEGG pathway within its carbohydrate metabolism. On the other hand, sucrose and beta-D-glucose are, instead, downregulated. Theoretically, this result correlated with Hornbuckle et al. [60], who explained two steps of starch hydrolyzation, i.e., rapid and slower steps. The rapid one results in maltose and maltotriose formation, and the slower one involves hydrolysis of maltotriose into maltose and glucose. The α-amylase is an enzyme produced by the pancreas that functions to catalyze the specific hydrolysis of α-1,4-glucosidic bonds of starch and glycogen. When α-amylase hydrolyze starch, this will produce the principal product with a predominance of maltose (α-1,4-glycosidic bond), isomaltose (α-1,6-glucosidic bond), and small amounts of glucose, which correlated with our result. Moreover, Hornbuckle et al. [60] also stated that the enzymes maltose and isomaltose are integral parts of the microvillus membrane, so that the final hydrolysis of these two compounds (maltose and isomaltose) occurs at the surface of the intestine mucosal cell. The result of the KEGG pathway of carbohydrate synthesis in the cecal content of FR20 group is the same as the result at FR40. This result indicated that, although FR was carried out, it did not interfere with the function and biochemical processes of the intestine, specifically in carbohydrate synthesis.

AICAR also downregulated AICAR in the cecum of the FR20 group. AICAR, Aminoimidazole-4-carboxamide-1-b-DD-ribofurano-side, is one of the AMPK activators. It is phosphorylated in cytosol by adenosine kinase and is further converted to AICAribotide (ZMP), which activates AMPK by mimicking AMP [61,62]. Hereafter, AMPK regulates the expression of various genes that are involved in the glucose metabolism [63]. This condition, related to carbohydrate synthesis, results in the feed restriction group’s cecal result. Our study showed that FR caused the AICAR to be downregulated; then, this condition resulted in a decrease in beta-D-glucose. So, it indicated that AICAR effected other physiological responses, such as hypoglycemia [64]. In addition, this study showed that dietary restriction provided another advantage with the decrease in histamine in the cecum of the FR40 group. Histamine, an amino acid metabolism, plays an important role in epithelial protection. Lower concentrations of histamine might be protecting the epithelial, whereas higher concentrations of it might be detrimental to epithelial protection from pathogen infection [65]. The continuously production of small amounts of histamine, which is produced by the gut microbiota, can lead to suppression of intestinal inflammation [66]. In the blood of laying hens of both FR20 and FR40 groups, two lipid metabolites (stearidonic acid and 13-L-Hydroperoxylinoleic acid) enriched fatty acid biosynthesis. This study indicated that FR conditions initiated in chickens at 48 weeks of age resulted in an increase in lipid metabolism. Above all, FR altered cecal and blood metabolic pathways, especially biosynthesis pathways of lipids and carbohydrates.

### 4.4. The Relationship of Different Relative Abundance of Bacteria in the Cecal Microbiota with Cecal and Serum Metabolites

Microbiota related to cecal metabolite (metabolomic) analysis in the FR20 group in this recent study showed that phylum Firmicutes have a positive correlation with metabolites such as LysoPC (18: 2 (9Z, 12Z)) (through involvement of the genus *Oribacterium*), L-Aspartic acid (involving the genus *Ruminiclostridium_9*, *Oribacterium*, *Butyricicoccus*), beta-D-Glucose (through involvement of the genus *Ruminococcaceae_UCG_004*, *Ruminococcaceae_UCG_014*), and AICAR (involving the genus *Ruminococcaceae_UCG_014*, *Ruminococcaceae_UCG_005*, *Lachnospiraceae_NK4A136_group*). This LysoPC (18: 2 (9Z, 12Z)) metabolite has an important role in the biosynthesis of fatty acids [44], L-aspartic acid and AICAR play a role in amino acid metabolism [61,62], and beta-D-Glucose functions in carbohydrate synthesis [67]. In the human intestine, Ruminococcaceae has a functional ability to degrade nondigestible carbohydrates such as resistant starch, hemicellulose, and cellulose [68].

Furthermore, compared to the FR20 group, phylum Fusobacterium was the only phylum in the FR40 group that had a positive correlation with oleic acid in chicken cecum and choline chicken serum. Oleic acid is a monounsaturated fatty acid with several biological functions: it enhances mitochondrial oxidation of saturated fatty acids (by increasing triacylglycerol and by reducing diacylglycerol and ceramide production) and displays the ability to prevent SFA-induced inflammation, thus protecting the cells from inflammation [69]. Choline, a micronutrient often classified with the B-vitamins, is a proven lipotropic agent in several species of animals [70]. A lipotropic agent is a compound that has an affinity for lipids and thus can help to catalyze the breakdown of fat during metabolism in the body [70,71]. Choline testing can be performed using a blood sample, called the plasma concentration of fat-soluble choline biomolecules [70]. All these FR40 correlations indicated that feeding restriction can affect phylum Fusobacterium, triggering both oleic acid for lipid synthesis and choline for the synthesis of vitamins, in cecum and blood, respectively.

## 5. Conclusions

Understanding the fundamentals of host–microbial interactions is crucial for creating cost-effective laying hen production techniques. There will undoubtedly be savings under quantitative FR circumstances owing to lower feed prices, particularly for hens in the late phase of life. As a result, this research aids in the identification of intestinal bacteria, the relationship between microbiota and metabolite profiles, as well as their metabolic pathways in connection to host nutritional needs and gut nutrient availability. This research may be utilized as a foundation for future research into how dietary interventions affect the intestinal microbiota, host physiology, egg quality, and feed efficiency in chickens. In the end, this method can be applied as an alternative method in late-phase laying hens to reduce overfeeding which aims to reduce feeding costs.

## 6. Patents

There is no patent resulting from the work reported in this manuscript.

## Figures and Tables

**Table 1:** Diet composition and calculated nutrition levels.

Ingredients (as Fed-Basis %)	Composition
Yellow corn grain	52.2
Soybean meal dehulled (CP 47%)	30.3
CaCO_3_	11.1
MonoCaP	2.2
Soybean oil	3.0
DL-Met	0.3
Salt	0.3
Vitamin premix ^1^	0.05
Mineral premix ^2^	0.05
NaHCO_3_	0.5
Metabolizable energy, kcal/kg	2953.2
Total	100
Nutrient (calculated)	
Crude protein (%)	18.1
Crude fat (%)	5.4
Calcium (%)	4.7
Available phosphorus (%)	0.6
Methionine + cysteine (%)	0.9

^1^ The vitamin premix provided the following (per kilogram of diet): vitamin A, 10,000 IU; vitamin D3, 2000 IU; vitamin E, 15 mg; vitamin K, 4 mg; thiamine, 2 mg; riboflavin, 6 mg; pyridoxine, 4 mg; vitamin B12, 0.02 mg; pantothenate, 12 mg; niacin, 40 mg; folate, 1 mg; biotin, 0.02 mg. ^2^ The mineral premix provided the following (per kilogram of diet): Zn, 90 mg; Mn, 100 mg; I, 1 mg; Cu, 15 mg; Fe, 90 mg; I, 200 mg; Se, 0.15 mg; Co, 0.25 mg.

## Data Availability

No new data were created or analyzed in this study. Data sharing is not applicable to this article.

## Supplementary Materials

The following are available online at https://www.mdpi.com/article/10.3390/ani11113043/s1. Figure S1: Species-accumulation curves analysis of the species number in these samples, Figure S2: Ratio of Firmicutes: Bacteriodetes in all of groups, Figure S3: The correlation between relative abundance of phylum level in the cecal of FR20 group and metabolites, Figure S4: The relative abundance of phylum level in the serum that correlated with metabolites in serum of FR20 group, Figure S5: The correlation between relative abundance of phylum level in the cecal of FR40 group and metabolites, Figure S6: The relative abundance of phylum level in the cecal that correlated with metabolites in serum of FR40 group, Table S1: Biomarker metabolites in cecum and serum.
