# Supplementary material for: The Effect of Feeding Restriction on the Microbiota and Metabolome Response in Late-Phase Laying Hens
Source: Animals (Basel). 2021 Oct 24;11(11):3043. doi: 10.3390/ani11113043 (PMC8614447; doi:10.3390/ani11113043)
Supplement: Supplementary file 1 [file animals-11-03043-s001.zip › Supplemental Figures_animals-1330041.pdf]

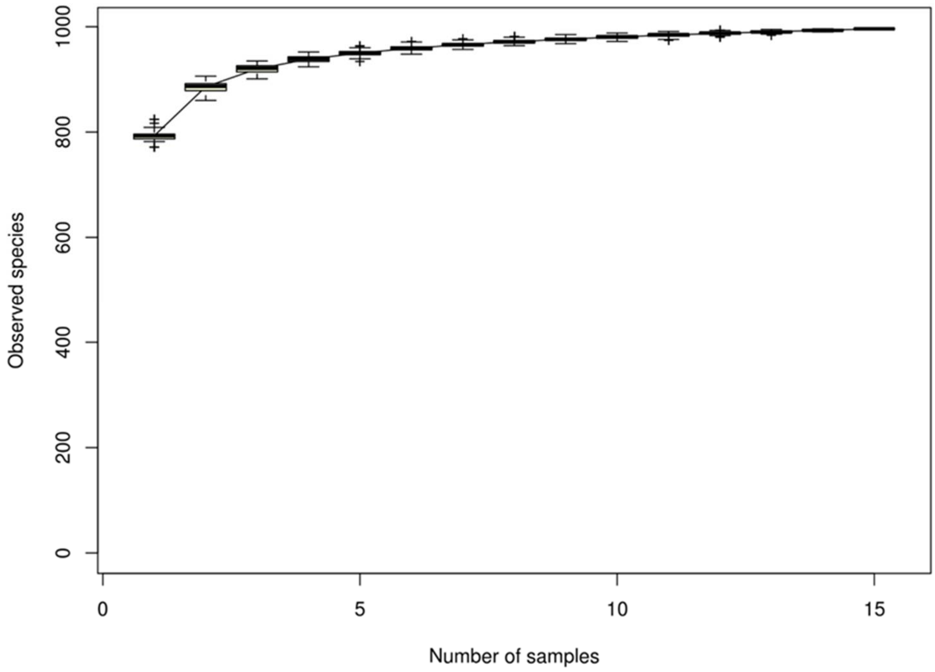

Supplemental Figure S1. Species-accumulation curves analysis of the species number in these samples. A large proportion of species existed in the cecal communities.

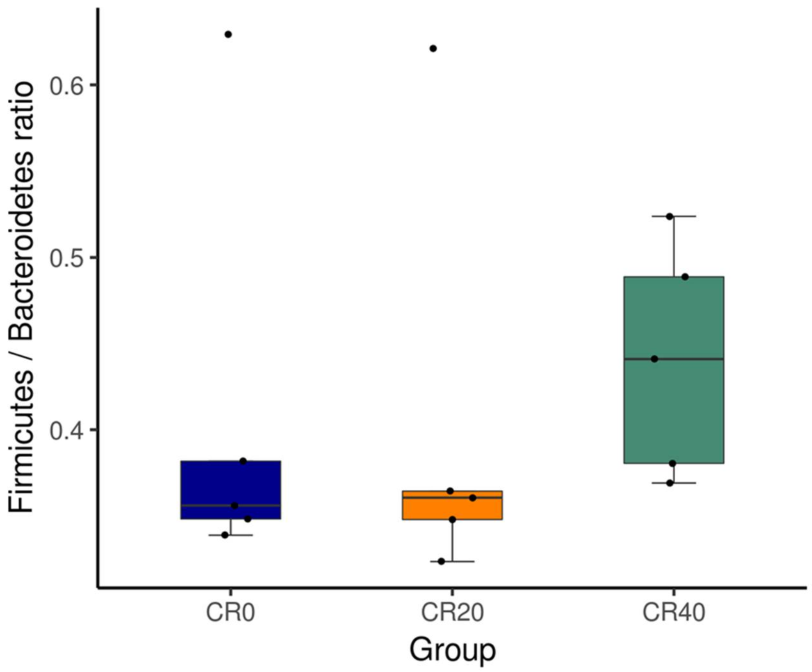

Supplemental Figure S2. Ratio of Firmicutes : Bacteriodetes in all of groups.

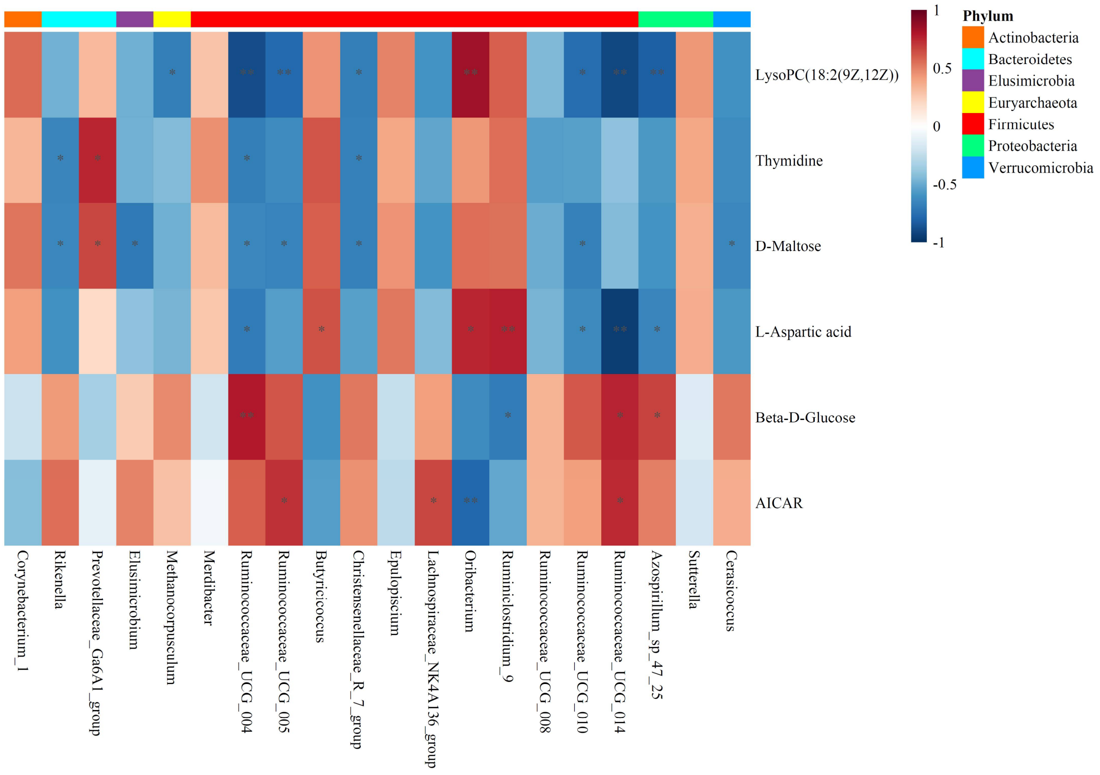

Supplemental Figure S3. The correlation between relative abundance of phylum level in the cecal of FR20 group and metabolites. Firmicutes was the high proportion of bacteria-correlated metabolites that had positively and negatively correlated in the phylum level, followed by Bacteriodetes, Proteobacteria, Elusimicrobia, Euryarchaeota, and Verrucomicrobia.

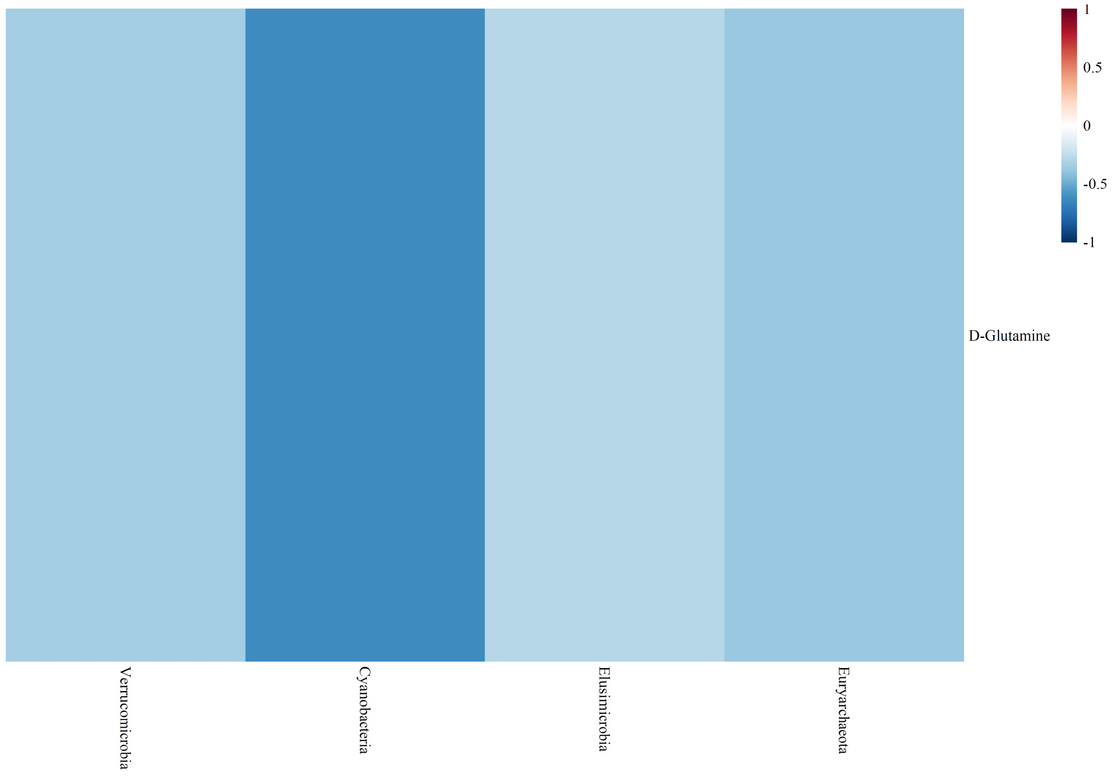

Supplemental Figure S4. The relative abundance of phylum level in the serum that correlated with metabolites in serum of FR20 group. No positively correlated found between cecal microbiota of FR20 group and serum metabolites.

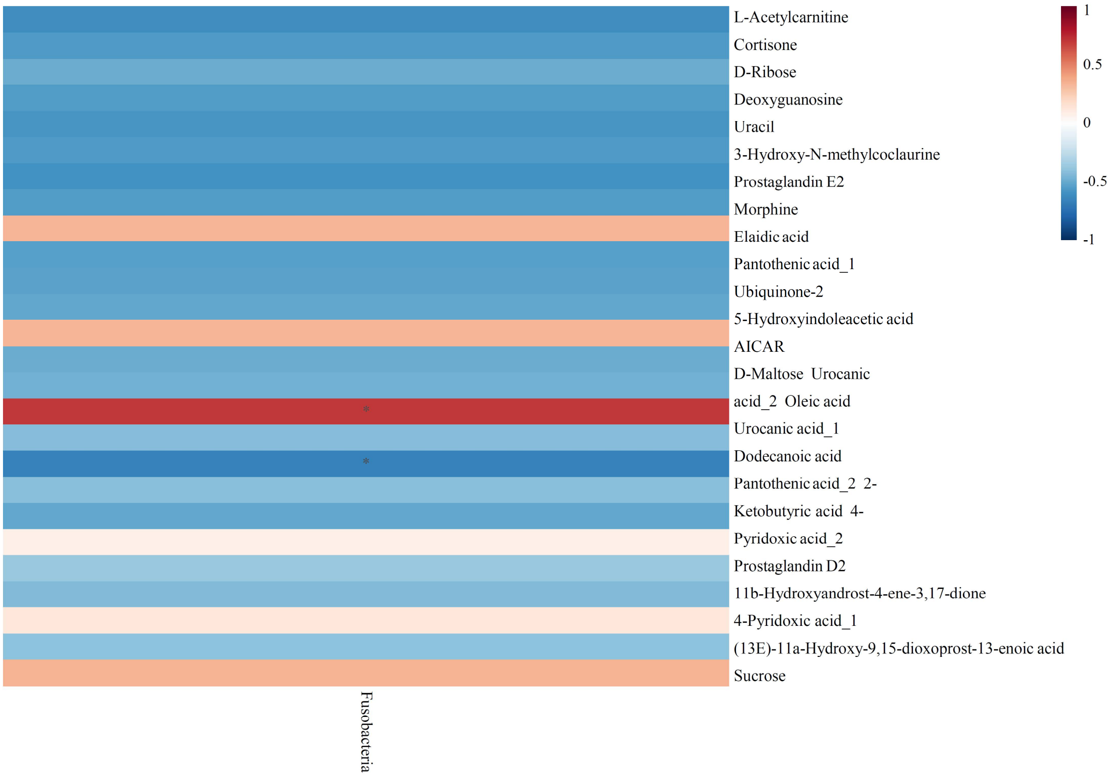

Supplemental Figure S5. The correlation between relative abundance of phylum level in the cecal of FR40 group and metabolites. Showed Fusobacterium had positively correlated with oleic acid and negatively correlated with Dodecanoic acid.

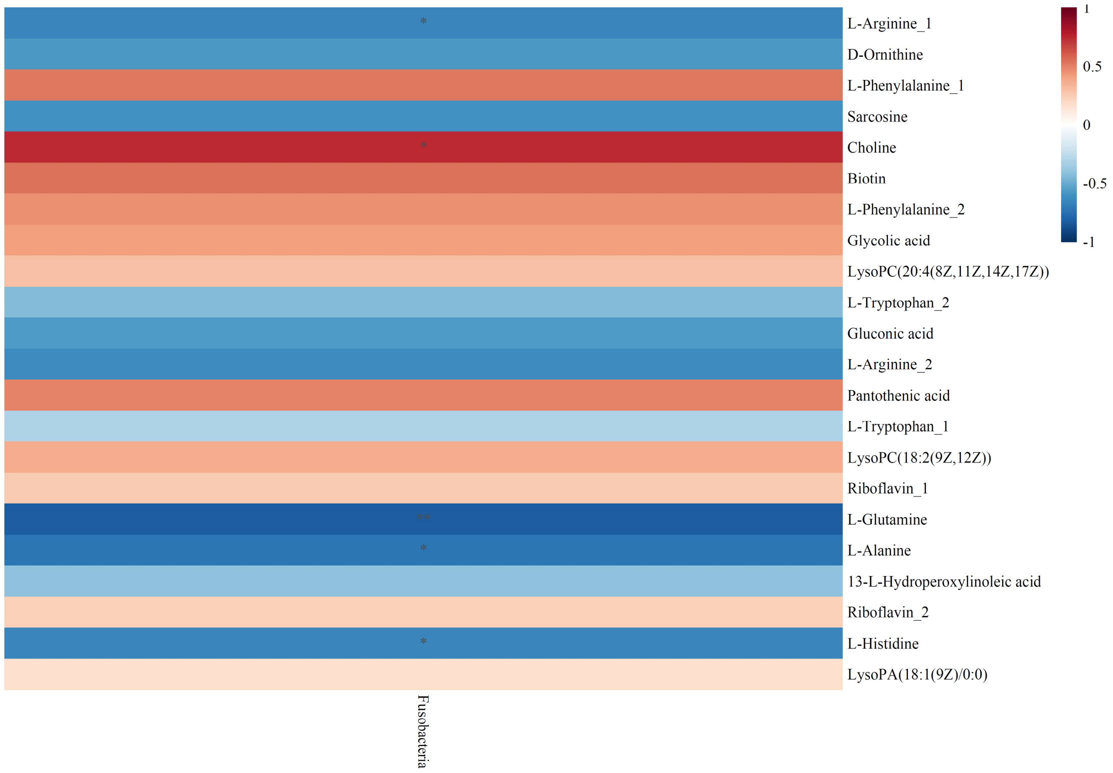

Supplemental Figure S6. The relative abundance of phylum level in the cecal that correlated with metabolites in serum of FR40 group. Fusobacterium had positively correlated with choline and negatively correlated with L-Glutamine, L-Arginine_1, L-Histidine, and L-Alanine.
